# Effect of Free Healthcare Policy for Children under Five Years Old on the Incidence of Reported Malaria Cases in Burkina Faso by Bayesian Modelling: “Not only the Ears but also the Head of the Hippopotamus”

**DOI:** 10.3390/ijerph17020417

**Published:** 2020-01-08

**Authors:** Mady Ouédraogo, Toussaint Rouamba, Sékou Samadoulougou, Fati Kirakoya-Samadoulougou

**Affiliations:** 1 Centre de Recherche en Epidémiologie, Biostatistiques et Recherche Clinique, Ecole de Santé Publique, Université Libre de Bruxelles, 1070 Brussels, Belgium; omadess@yahoo.fr (M.O.); rouambatoussaint@gmail.com (T.R.); 2 Institut de Recherche Santé et Sociétés, Faculté de Santé Publique, Université catholique de Louvain, 1200 Brussels, Belgium; 3 Institut National de la Statistique et de la Démographie (INSD), Ouagadougou 01 BP 374, Burkina Faso; 4 Unité de Recherche Clinique de Nanoro, Institut de Recherche en Sciences de la Santé, Centre National de la Recherche Scientifique et Technologique, Ouagadougou 11 BP 218, Burkina Faso; 5 Evaluation Platform on Obesity Prevention, Quebec Heart and Lung Institute, Quebec, QC G1V 4G5, Canada; osekou@gmail.com; 6 Centre for Research on Planning and Development (CRAD), Université Laval, Quebec, QC G1V 0A6, Canada

**Keywords:** Burkina Faso, free-of-charge health care, malaria, Bayesian, maps, spatiotemporal

## Abstract

Burkina Faso has recently implemented an additional strategy, the free healthcare policy, to further improve maternal and child health. This policy targets children under five who bear the brunt of the malaria scourge. The effects of the free-of-charge healthcare were previously assessed in women but not in children. The present study aims at filling this gap by assessing the effect of this policy in children under five with a focus on the induced spatial and temporal changes in malaria morbidity. We used a Bayesian spatiotemporal negative binomial model to investigate the space–time variation in malaria incidence in relation to the implementation of the policy. The analysis relied on malaria routine surveillance data extracted from the national health data repository and spanning the period from January 2013 to December 2018. The model was adjusted for meteorological and contextual confounders. We found that the number of presumed and confirmed malaria cases per 1000 children per month increased between 2013 and 2018. We further found that the implementation of the free healthcare policy was significantly associated with a two-fold increase in the number of tested and confirmed malaria cases compared with the period before the policy rollout. This effect was, however, heterogeneous across the health districts. We attributed the rise in malaria incidence following the policy rollout to an increased use of health services combined with an increased availability of rapid tests and a higher compliance to the “test and treat” policy. The observed heterogeneity in the policy effect was attributed to parallel control interventions, some of which were rolled out at different paces and scales. Our findings call for a sustained and reinforced effort to test all suspected cases so that, alongside an improved case treatment, the true picture of the malaria scourge in children under five emerges clearly (see the hippopotamus almost entirely).

## 1. Introduction

Malaria is an infectious disease caused by a parasite of the *Plasmodium* genus. It is transmitted to humans by the bite of an infected mosquito, the female *Anopheles*. It is a potentially fatal disease in the absence of early diagnosis followed by effective treatment [1]. Sub-Saharan Africa is paying the heavy burden of this disease, with children younger than 5 years and pregnant women being the most vulnerable groups [1,2]. With international mobilization over the past 15 years, there was a significant reduction in the burden of this disease [2,3,4]. This mobilization has made it possible to implement multifaceted control strategies (prevention, case management, and epidemiological surveillance) that were adopted by all malaria-endemic countries [5,6]. Despite all these efforts, the malaria burden remains very high in six sub-Saharan African countries, including Burkina Faso [1,7]. Several reasons were given to justify the persistence of the burden. These are mainly inaccessibility to health care, insufficient diagnosis, and early and effective management of malaria cases in health facilities and at the community level [8,9]. These latter factors, combined with a high prevalence of self-medication [10,11] and low health service utilization [8,12], among others, contribute to an underestimation of the malaria burden in the population and under-reporting by the surveillance system. Indeed, among the real cases of malaria, only a small proportion, qualified as the “hippopotamus ear”, are diagnosed (clinical and/or biological) and reported by the health facilities [13].

In a resource-constrained context, WHO advocates that countries establish a free-of-charge care policy as an effective way to expand the coverage and use of health services by vulnerable (poor) populations. This strategy was implemented in 20 countries in Sub-Saharan Africa [14]. However, there is little scientific evidence on the impact of free-of-charge health care on children and malaria morbidity.

Burkina Faso has recently implemented several programs to address this ongoing health concern. These include the systematic use of rapid diagnostic test (RDT) in malaria diagnostic (in 2013), integration of malaria data into the Health Information System 2 (DHIS2), results-based financing (in 2014), seasonal chemoprevention campaigns (in 2014), decentralization of health care through the 130 Communes project (in 2015), use of community-based health workers (in 2016), and free-of-charge health care for children younger than 5 years (in 2016). These measures should enable health facilities to diagnose all true cases of malaria (see the hippopotamus almost entirely) in a comprehensive way [15] and treat them effectively (decrease the progression of cases to adverse outcomes, i.e., death). This is achieved by significantly improving the population’s access to health care, as well as the availability of diagnostic methods and the promptness and completeness of data reporting (improvement of the routine surveillance system).

This improvement in the routine surveillance system should provide decision-makers with reliable estimates of the malaria burden for control planning. Unfortunately, the current use of routine data is limited to estimating gross rates for reporting purposes in the statistical yearbook [7,16]. Such an analysis has enormous shortcomings, as the data have particular characteristics that limit the use of traditional statistical methods. Indeed, these analyses do not take into account subnational heterogeneity and disparities and fail to assess the effects of interventions on the spatial and temporal evolution of the malaria burden. Bayesian hierarchical spatio-temporal models have recently been developed, validated, and applied to malaria data to produce robust and reliable estimators [17,18,19,20,21]. These models have proven useful in overcoming the problems of data complexity [21,22,23,24].

Here, we present the results of a model to evaluate the impact of malaria control interventions in Burkina Faso’s 70 health districts (operational entities of the health system). The model was used to assess the impact of the “free-of-charge health care among children younger than 5 years” program on the incidence of malaria cases (suspected, tested, and confirmed) in health districts between 2013 and 2018. This provides a new framework for rigorously evaluating malaria control programs at the health district level, as well as child health monitoring programs in general, which are based on surveillance data, controlling confounding factors while taking into account the inherent correlation of these types of data over time and space.

## 2. Materials and Methods

### 2.1. Setting

This research used routine data from all 70 health districts of Burkina Faso. The population at risk of malaria in these 70 districts was estimated at about 20,244,080 in 2018, ranging from 141,830 in the district of Nanoro to 285,922 in the district of Gaoua [25]. Health districts in the southwestern region are characterized by abundant rainfall (more than 950 mm/year) while those in the northern region are typically drier (599–715 mm/year). Malaria transmission is holoendemic in the Gaoua health district; hyperendemic in the districts of Boromo, Toma, Batié, Dano, and Diébougou; and mesoendemic in the other districts [26]. The climate factors are favorable to the creation and maintenance of breeding sites, leading to the perpetuation of the life cycle of female *Anopheles* mosquitoes, the vectors of malaria. In Burkina Faso, *P. falciparum* is responsible for 90% of all infections and the rest is due to *P. malariae* and *P. ovale* [27]. The entomological inoculation rate (EIR) is estimated at about 192 infective bites/person (2011) and the major contributor was *An. Gambiae s.s* (70%), *An. Funestus*, *An. Coluzzii*, and *An. coluzzi* [27].

### 2.2. Data Sources

#### 2.2.1. Malaria Data for Children Younger than 5 Years

Data on malaria cases (suspected, tested, confirmed) were extracted from Burkina Faso’s health information system (ENDOS-BF) [28]. A total case of malaria was defined as any case classified as malaria in the outpatient registry, whether or not it was parasitologically tested. A tested case was defined as a suspected case of malaria that was parasitologically tested. A confirmed case of malaria was defined as a case where malaria was diagnosed parasitologically, either by microscopy or rapid diagnostic test (RDT). Non-malarial cases refer to all cases of disease in children under five diagnosed in health centers (other cause of disease than malaria). It was obtained by subtracting the total number of malaria cases diagnosed in children under five from the total number of consultations of children under five.

The data cover the period from January 2013 to December 2018 and were reported for children younger than 5 years. ENDOS-BF was implemented in 2013 and has significantly improved data transmission timelines and information completeness. Indeed, the coverage rates of the health indicators and in particular those relating to malaria have improved considerably (confirmation rate of malaria cases was estimated at 91.7% in 2018 compared to 62.4% in 2013) [29]. In addition, the concordance index was improved and was estimated at 61% in 2013 and 80% in 2018 [30].

The monthly malaria incidence (suspect, tested, and confirmed) was estimated by dividing the total number of monthly malaria cases by the number of children younger than 5 years per health district. Monthly malaria incidences in the district were calculated to better assess the temporal trend and seasonal fluctuations in malaria incidence.

The population of children by health district from 2013 to 2018 was estimated using the population projections made in 2006 as part of the General Population and Housing Census in Burkina Faso, adjusted for the annual population growth rate [25].

#### 2.2.2. Intervention Coverage Data

Data on malaria intervention programs, namely, insecticide-treated nets (ITNs) and case management with artemisinin-based combination therapies (ACTs), as well as health care sought in the case of fever among young children, were obtained from the National Malaria Indicator Survey (MIS) conducted in 2014 and 2017 [31,32]. Due to the low coverage of indoor residual spraying in most health districts as a malaria prevention strategy, this variable was not included in the analysis. The 2014 and 2017 MISs were cross-sectional surveys conducted by the National Institute of Statistics and Demography (INSD) in collaboration with ICF International among a representative sample of 6552 households in 2014 and 6322 households in 2017. The sample for this survey is representative of the regional administrative subdivision, based on a two-stage stratified cluster survey.

A socio-economic variable at the district level was defined as the proportion of the poorest households (first quintile of wealth; MIS 2014, MIS 2017). Information provided by mothers or caregivers was used to calculate the proportion of children younger than 5 years with fever in the 2 weeks prior to the interview who took any antimalarial drug, the percentage who took ACT, and the proportion of children younger than 5 years who had fever, for whom advice or treatment was sought.

The indicator of free-of-charge health care among children younger than 5 years was obtained by constructing a binary variable that takes the modality 0 for the period before 2016, the year of implementation of this policy by the Burkinabe State (2013–2015), and 1 for the period after 2016 (2016–2018).

#### 2.2.3. Data on the Availability of the Rapid Malaria Test (RDT) from Health Facilities

The availability of rapid malaria tests (RDTs) in health facilities in Burkina Faso was obtained from the national survey on the performance of health facilities (SARA) conducted in 2012, 2014, and 2016 [33]. The sample for this survey is representative of the regional administrative subdivision, based on a two-stage stratified cluster survey.

#### 2.2.4. Environmental Drivers of Malaria Risk

Environment variables are likely to affect malaria transmission through their impact on vector populations. For this reason, the effects of the environment on malaria transmission are expected to appear with a delay associated with the life cycle of the vector [34,35].

The monthly average temperature and the cumulative rainfall data for each district were extracted from the Global Climate Data website (www.worldclim.org/) with a 1-km spatial resolution. The relative humidity data from the period January 2013 to December 2017 for each district were obtained from NASA (MERRA-2) [36]. These covariates have been widely used in mapping malaria in sub-Saharan Africa [3,17,18,21,26,37,38].

### 2.3. Statistical Analysis of Malaria

#### 2.3.1. Crude Incidence of Malaria in Children and Description of Covariates

Time series graphs were used to describe the inter-and intra-annual variations in malaria incidence during the study period (2013–2018). Short-term fluctuations were smoothed to better interpret long-term trends using a monthly “Hodrick Prescott filter” smoothing parameter [39]. In addition, lagged variables for time-varying climate predictors (precipitation, temperature) were used to take into account the malaria transmission cycle, suggesting that there is a period when the climate is suitable for malaria transmission and case occurrence [34]. Three different lag-times variables were constructed for each climate factor by averaging its values over the following periods: The current month and the previous month (lag 1), the current month and the two previous months (lag 2), and the current month and the three previous months (lag 3). Categorical variables were generated to take into account the distribution of variables because the relationship between malaria and environmental predictors is not always linear [40].

#### 2.3.2. Selection of Bayesian Covariates

The selection of Bayesian variables implemented in the spatio-temporal model was applied to identify the most important ITN coverage indicator and the delayed climate factor with their functional form (i.e., linear or categorical). Several binomial negative spatial-temporal models were generated with each of the variables (ITN) to determine which one best contributed to predicting reality. The ITN variable with a low deviance information criterion (DIC) was selected for inclusion in the models. In this study, six ITN coverage indicators, corresponding to the three indicators of possession and three indicators of use defined by Roll Back Malaria (RBM), were tested. As mentioned above, four variables, including no lag (lag 0) and lags (1–3), were constructed and then included in the model and the same process was used to select those to be included in the models according to value of DIC.

Data on interventions and the proportion of the poorest households provided by MIS (2014 and 2017) and SARA (2012, 2014, and 2016) summarized at the district level do not provide reliable coverage estimates, as the survey is designed to produce reliable estimates at the national and regional levels. Therefore, district coverage was estimated using binomial models implemented in a Bayesian framework on aggregate intervention and poverty data. Malaria cases observed in health facilities in Burkina Faso represent only a fraction of the total number of cases due to poor behavior in the use of health facilities [41]; the model was therefore adjusted to take into account the proportion of fever care-seeking behaviors among children reported in the 2014 and 2017 MISs. The proportion of fever care-seeking behaviors among children was also estimated at the district level using a conditional autoregressive model implemented in a Bayesian framework [42]. Details of the modelling of estimates at the district level are available in Appendix A.

#### 2.3.3. Statistical Modelling of Malaria in Children

A negative binomial spatial-temporal negative model implemented in a Bayesian framework was applied to malaria data (tested and confirmed) for children to describe the spatio-temporal evolution of malaria incidence at the district level and to determine the effect of free-of-charge health care among children younger than 5 years of age. The heterogeneity of the impact was considered via year-specific random effects and spatially structured and unstructured random effects. The random effects were modelled at the district level via Gaussian distributions with non-informative distribution parameters for time and a conditional autoregressive prior [42] model for space. The space–time interaction was included to take into account the spatial variation of malaria from one month to the next. In addition, the temporal correlation over several months was captured by monthly random effects modelled by a first-order autoregressive process.

The “integrated nested Laplace approximation” (INLA) package implemented in R was used to obtain posterior mean distribution of the marginal effect of model parameters and the effect of interventions. These parameters were summarized using the incidence rate ratio (IRR) and the 95% credible interval.

Details of the modelling of estimates at the district level are available in Appendix B.

### 2.4. Ethics Approval

This study used data from health facilities reported in the DHIS2 and made accessible by the Directorate General of Studies and Sectoral Statistics of the Ministry of Health. The ethics approval was waived because data analysis was carried out at a district level with no reference to individual-level identification particulars.

## 3. Results

### 3.1. Summary of Crude Estimates Incidence

During the study period, the annual incidence of malaria (suspected, confirmed) per 1000 children under five increased. Indeed, it increased from 1997 cases (95% CI: 1995, 1999) in 2013 to 4243 cases per 1000 (95% CI: 4241, 4245) in 2018 for suspected cases and from 1204 cases per 1000 (95% CI: 1203, 1205) in 2013 to 2933 cases per 1000 (95% CI: 2932, 2935) for confirmed cases. Number of confirmed cases increased from 11,087,535 in 2013–2015 to 1,569,005 in 2016–2018, an increase of 42%.

**Figure 1:**
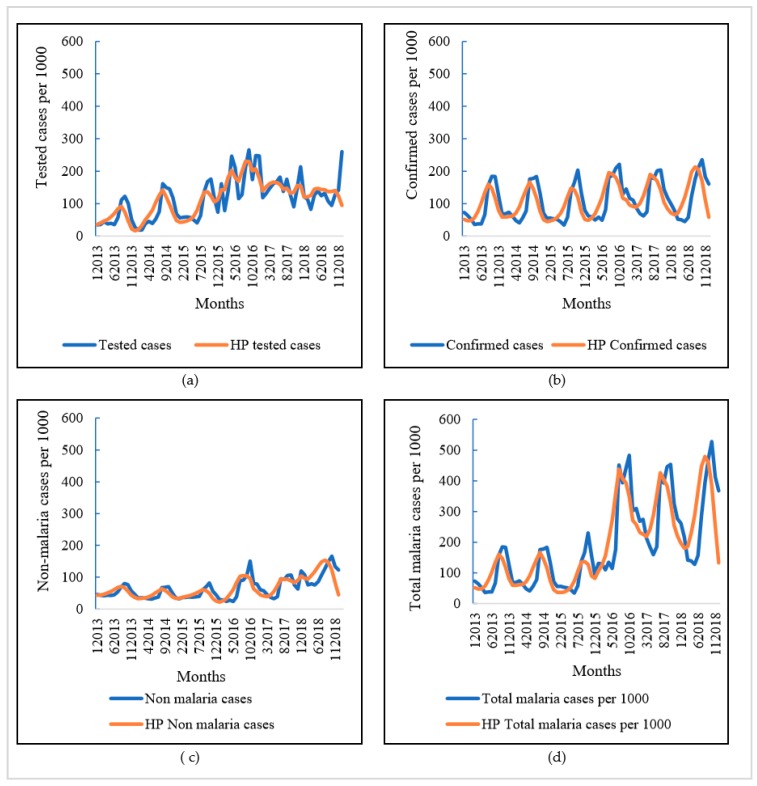
Trends of crude incidence of malaria reported cases over 72 months (**a**) tested incidence and (**b**) confirmed incidence (**c**) non-malaria cases, and (**d**) total cases. HP: Hodrick Prescott. The x-axis represents the time that combines both the month and the year. The last four digits represent the year and the first digits represent the month and (**a**) y: number of malaria tested cases per 1000 children under five (**b**) y: number of malaria confirmed cases per 1000 children under five (**c**) y: number of non-malaria cases per 1000 children under five (**d**) y: number of total malaria cases per 1000 children under five.

The monthly description of malaria cases per 1000 children under five is presented in Figure 1. Overall, the number of malaria cases tested and confirmed monthly per 1000 children increased between 2013 and 2018, with an interannual variation. Thus, the period from July to December is the time of the year in which children are most affected. In addition, similar to the incidence of confirmed cases, malaria suspected cases and non-malaria cases also showed an upward trend over the same period with a peak from 2016 onwards. This increase in the incidence of malaria was greater after 2016 and coincides with the implementation of the free-of-charge health care policy for children under five.

**Figure 2:**
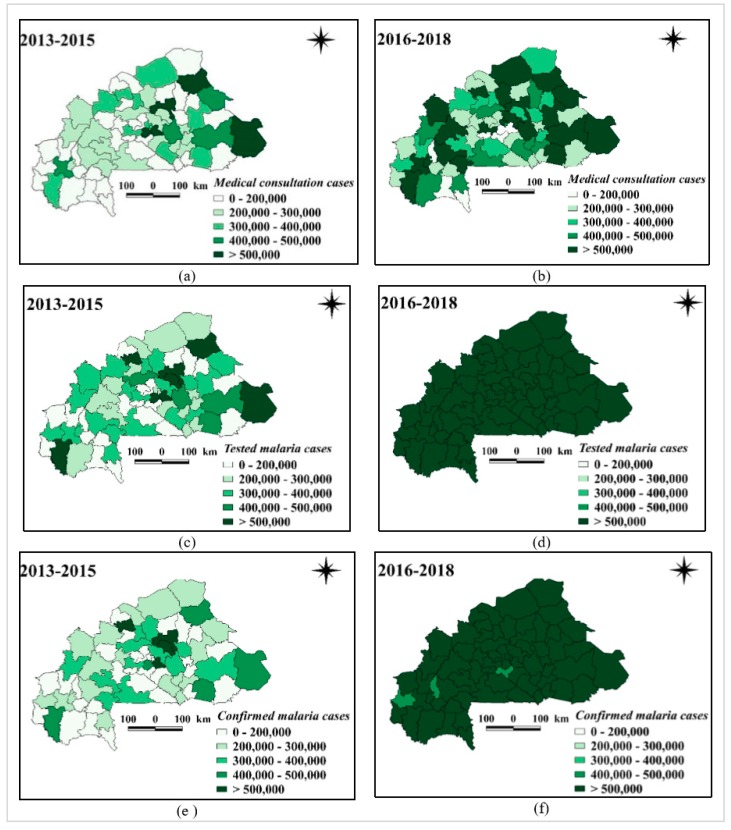
Spatio-temporal distribution: (**a**) number of consultations per district 2013–2015; (**b**) number of consultations per district 2016–2018; (**c**) number of cases tested per district 2013–2015; (**d**) number of cases tested per district 2016–2018; (**e**) number of confirmed cases per district 2013–2015; (**f**) number of confirmed cases per district 2016–2018.

Aggregate cases at the level of the 70 health districts (all consulted cases, suspected cases, and confirmed cases) were evaluated, comparing the average monthly cases before and after the adoption of the national policy of free-of-charge health care for children younger than 5 years of age. All indicators (all consultation cases, tested, and confirmed malaria cases) increased significantly between the pre- and post-intervention periods in all health districts. However, the incidence of malaria (suspect, confirmed) and the number of visits varied considerably between districts and the two periods (Figure 2a–f).

### 3.2. Results of the Bayesian Variable Selection and Best Model

The results of the Bayesian variable selection of ITN coverage indicators show that the proportion of children younger than 5 years of age who spent the night under a mosquito net had the lowest DIC among all ITN indicators. Therefore, this indicator was used as a measure of ITN coverage. The mean values of categorical climate variables, lagging by 1 month for temperature and rainfall, had the lowest DIC.

### 3.3. Results of the Space–Time Modelling

Table 1 presents the spatio-temporal estimates of the effects of interventions adjusted for climatic and socio-economic confounders. The Besag York and Mollié (BYM) model with a spatially and temporally structured component (AR1) was the best model to predict the incidence of malaria in children. Indeed, the DIC was 83,408 compared to the model formulated in BYM with a spatially and temporally structured component (AR1), for which the DIC was 83,068.

#### 3.3.1. Effects of Interventions on Malaria Incidence

The implementation of the free-of-charge policy was significantly associated with the incidence of both tested and confirmed cases. Indeed, taking into account the confounding factors, the introduction of free care was associated with an increase in the number of cases tested and confirmed, i.e., twice as many as the period before free care. In addition, seeking care for child fever and the availability of RDTs were positively associated with confirmed and tested malaria cases (Table 1). Thus, a 100% increase in the proportion of mothers who sought care for their children who suffered from fever was associated with a 1.5-fold increase in the number of confirmed and tested cases while a 100% increase in the availability of RDTs in health centers was associated with more than 1.5-fold increase in the number of tested and confirmed cases.

ITN and ACT coverage had a protective effect even though they were statistically not significant. Indeed, a 100% increase in the proportion of children sleeping under an ITN was associated with a 31% decrease in malaria incidence, and a 100% increase in the proportion of fevers treated with ACT was associated with a 10% decrease in incidence.

Temperature and rainfall were not significantly associated with the malaria indicators studied in this research.

#### 3.3.2. Spatial, Temporal, and Spatio-Temporal Variation in Malaria Incidence

Over the study period, the temporal variability of confirmed (0.298, 95% BCI: 0.168, 0.565) and tested (0.265, 95% BCI: 0.149, 0.492) cases was higher than the spatial variability of confirmed (IRR: 0.004, 95% BCI: 0.001, 0.014) and tested (0.004, 95% BCI: 0.000, 0.016) cases. The temporal variability of confirmed cases was slightly higher than that of tested cases. The space–time interaction effect was significant for confirmed cases (IRR: 0.121, 95% BCI: 0.095, 0.154) and for tested cases (IRR: 0.116, 95% BCI: 0.089, 0.148), indicating that malaria incidence was heterogeneous across districts during the period (Table 1).

**Figure 3:**
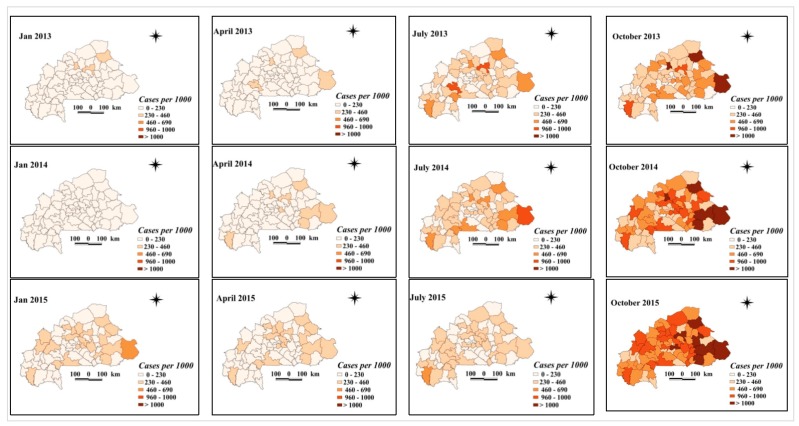

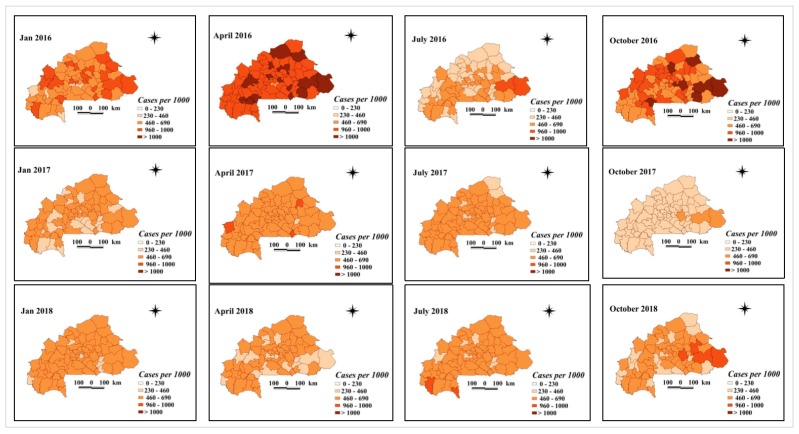
Space–time patterns of tested malaria incidence (cases per 1000 children) in children less than 5 years estimated from the Bayesian spatio-temporal model.

**Figure 4:**
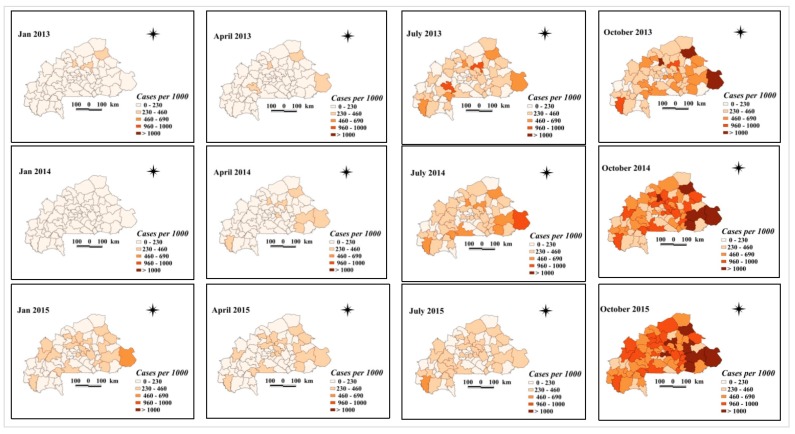

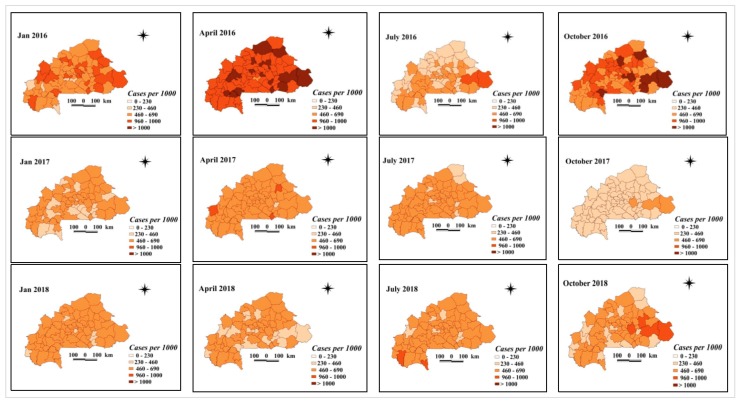
Space–time patterns of confirmed malaria incidence (cases per 1000 children) in children less than 5 years estimated from the Bayesian spatio-temporal model.

**Figure 5:**
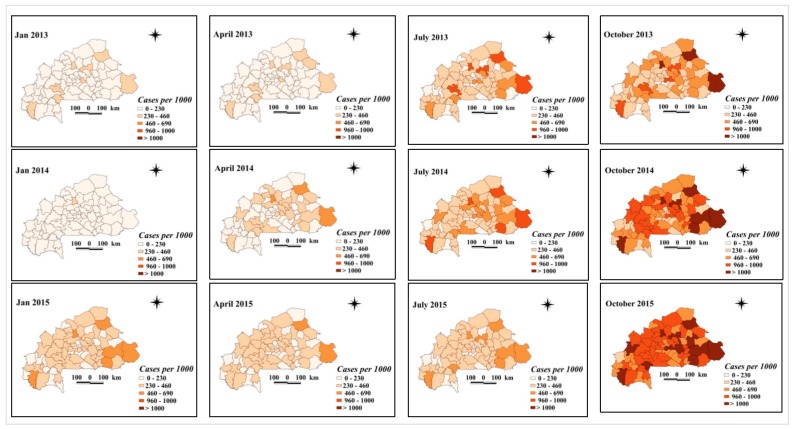

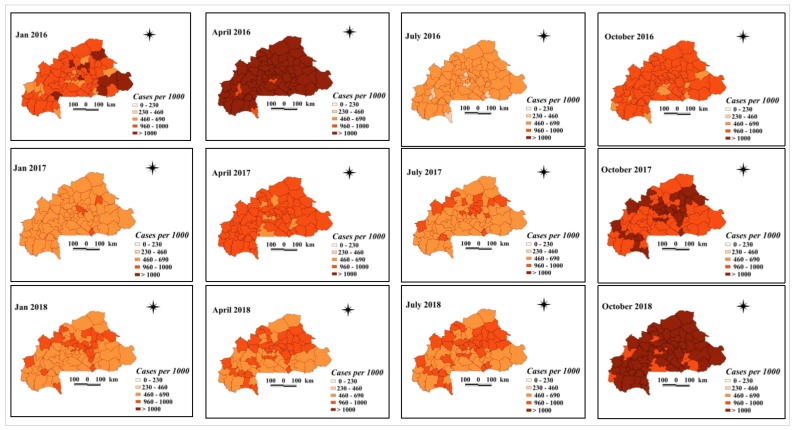
Space–time patterns of confirmed malaria rate (confirmed cases per 1000 total cases) in children less than 5 years estimated from the Bayesian spatio-temporal model.

Maps of smoothed malaria incidence estimated from Bayesian models are presented in Figure 3 and Figure 4 for the first month of each quarter and study year (January, April, July, and October). The number of malaria cases tested and confirmed differed between the pre- and post-intervention periods. In 45 of the 70 health districts, there was an increase, with at least twice as many confirmed malaria cases as before the implementation of the free health care policy in 2016. Districts with a sharp increase in the number of confirmed malaria cases per 1000 after the intervention period were located in the central, north-central, southwestern, upper basins, and west-central regions. These are the districts of Barsalogho, Baskuy, Kampti, N’Dorola, and Sabou (Figure 4), where the number of confirmed malaria cases per 1000 exceeded the number of cases by 4 times before free care was introduced. In 52 of the 70 health districts, the number of tested malaria cases per 1000 increased by at least twice as much compared with before the implementation of the free policy in 2016. This increase was higher in the Baskuy, Kampti, N’Dorola, Sabou, and Sapone districts, where the number of cases tested after the intervention was at least four times higher than the number of cases tested before the intervention (Figure 3). The number of malaria cases confirmed (among the total cases) was different between the pre- and post-intervention periods (Figure 5). Out of 70 health districts, there was an increase in 17 health districts (two-fold increase). Districts with a substantial increase in the number of confirmed malaria cases after the intervention period were Barsalogho, Baskuy, Batié, Boussé, Dandé, Diébougou, Kampti, Karangasso—vigué, Léna, Mani, N’dorola, Nongr-massoum, Pô, Sabou, Sapone, Sig-nonghi, and Thiou.

## 4. Discussion

In this study, we used routinely collected data to explore, firstly, the spatio-temporal pattern of malaria (suspected, tested, and confirmed) between 2013 and 2018, and secondly, to assess the effect of the free-of-charge health care policy on malaria reported cases incidence in Burkina Faso. Most studies in this field focused on the effects of interventions, such as ITNs, malaria diagnosis, and treatment, and our study is the first to provide evidence on the associations between the free-of-charge health care policy for children under five and malaria morbidity [15,38,43,44,45].

The main finding is the monthly increase in the number of confirmed malaria cases, especially from 2016 onwards. The hypothetical underlying factors that could explain this increase in confirmed malaria cases are discussed below.

Our findings show that the increase in the number of confirmed malaria cases is most likely the result of the strengthening of specific components of the health system in recent years, particularly the free-of-charge health care that led to an increase in the number of visits among children. This increase in the number of confirmed malaria cases could be also explained by the strengthening of diagnostic and case management capacities, combined with improved registration and reporting practices [15]. Indeed, the number of consultations (health facility attendance) as well as that of the tested and confirmed cases increased considerably after 2016 [7]. The increase in the number of confirmed malaria cases would therefore mean that cases were significantly underreported before 2016 and that the number of correctly diagnosed cases increased.

The free-of-charge health care policy for children under five, number of mothers seeking care for feverish children, and the availability of RDTs in health facilities were significantly associated with the number of tested and confirmed cases. Taking into account confounding factors, the implementation of the free-of-charge health care policy was significantly associated with an increase in the number of tested and confirmed cases. Indeed, both the number of tested and confirmed cases doubled with the implantation of the policy. The increases in both the number of tested and confirmed cases are associated with the implementation of the “test and treat” policy adopted in 2013. The latter intervention improved the availability of RDTs at the community level [8,9]. The effect of the introduction of malaria RDT on the number of tested and confirmed cases is in line with the result of a study carried out in the Democratic Republic of the Congo in 2017 [15].

Our results are also consistent with the recent trends in malaria morbidity. Indeed, a global increase in reported malaria cases has been reported recently that was linked to the improvement of the efficiency of routine surveillance systems [46]. Concomitantly, more countries are adopting a free-of-charge policy for vulnerable populations, including children [14]. However, there might still be an underreporting of malaria cases due to other barriers to health services, such as geographic accessibility, health care indirect costs, and socio-cultural barriers.

Similar studies in sub-Saharan Africa assume an under-reporting of malaria cases before the introduction of the free-of-charge health care policy and intensified diagnostic testing. For example, an increase in confirmed malaria cases was observed in Malawi between 2000 and 2010 [47], in Uganda between 1999 and 2009 [48], in Kenya between 2003 and 2009 [49], and in Togo between 2005 and 2010 [50]. These studies also indicate that improved access to malaria control interventions (subsidized treatment, community-based health service delivery), capacity building for health professionals, and an improvement of the reporting system have contributed to an increase in confirmed malaria cases [50]. Therefore, it can be assumed that the number of reported malaria cases has not yet reached the actual number of cases in Burkina Faso.

Our results also suggest that ITN and ACT coverage had a protective effect though not being statistically significant. Our results are consistent with those of other studies in sub-Saharan Africa that have shown that scaling up preventive interventions, such as ITNs and ACTs, leads to a reduction in malaria incidence. Indeed, some progress was made in reducing malaria morbidity and mortality through these interventions in Uganda [15,38,43,44,45], Senegal [51], Rwanda [52], Zambia [53], and Ghana [54]. Nevertheless, confirmed malaria cases would continue to increase with free-of-charge care and increased use of RDTs to test for febrile disease in children under five, and with the proportion of mothers seeking care for fever increasing over the 2015–2017 period [1].

The significant increase in the number of confirmed malaria cases reported in the health districts located in the central, north-central, south-western, Haut Bassin, and west-central regions could be attributed to disparities in access to health services. Indeed, in 2014, the proportion of mothers of children who suffered from fever and for whom treatment was sought at a modern health center was only 45.6% in the south-western region [31]. After the adoption of the free-of-charge health care policy for children under five, this proportion increased to 81.5% for the same region [32]. For the other regions mentioned above, this proportion increased by an annual average of 31% over the period 2014–2017 [32].

In addition, the disparities between health districts observed in the present study could be partly explained by other health programs. Indeed, prior to the implementation of the free-of-charge healthcare policy, other malaria control interventions were already ongoing in the health districts. Some of them had reached full coverage while others were being rolled out gradually. The community-based health workers program was implemented in 2015 countrywide while the seasonal malaria chemoprevention (SMC) strategy was progressively implemented from 2014 in 7 health districts to 65 districts in 2018 before reaching full coverage in 2019. The effectiveness of SMC on malaria risk reduction was reported in other studies in the central [55] and central-north regions [56] of Burkina Faso. Community-based interventions were reported to increase the coverage of most malaria control interventions [57]. Besides, though its coverage is limited, indoor residual spraying with long lasting insecticides, interrupted in 2012, was resumed in 2017 in three health districts, and there is evidence for its capacity to reduce malaria morbidity at the community level [58].

Spatially unstructured heterogeneity was much higher than spatially structured variability, which may imply high endemicity across the country, regardless of geographical location. The temporal variation was higher than the spatial variation, reflecting the stronger influence of seasonality in malaria transmission, due to meteorological variability. Similar results have been used to develop forecasting models in Burundi [59] and Ethiopia [34]. However, it is interesting to note that the seasonal structure of malaria incidence varies from country to country, which supports the evidence of a complex relationship between meteorological factors and malaria transmission and the need for regionally adapted forecasting models.

Temperature and precipitation were not significantly associated with malaria in this study. Studies on malaria carried out in Burkina Faso at the local level (Commune of Nanoro and Ouagadougou) assessing the temporal relationship between weekly rainfall and temperature data and weekly malaria cases showed a significant association [60,61]. The findings in our study could be partly explained by the type of data used to assess this relationship. Moreover, our results give an overview of this relationship at the national level.

Our study has some limits. The routine data we used in our study are known to be subject to biases. The use of confirmed malaria cases as the outcome, the integration of covariates in the model, and the inclusion of the population as a denominator helped to limit the bias associated with the use of routine data in the estimation of model parameters [58]. In addition, the CAR models could be subject to estimation biases due to ecological error [62]. However, the modeling framework used made it possible to take into account the uncertainty in the estimates, and biases due to the various data sources, which made it possible to obtain robust estimates. Another limit of our study is that the variable “ACT use” might be overestimated due to some suspected cases tested as negative but receiving ACT anyway. However, we expect this bias to have a moderate to low effect on our estimates. Indeed, a study conducted in Burkina Faso evidenced a shift in health workers’ attitudes when faced with negative RDT; they tended to prescribe antibiotics rather than ACT. The authors found that 92.89% of nurses were compliant with the rapid test result [63]. At a regional level, a multi-country study conducted in three West African countries, including Burkina Faso, showed an overall noncompliance rate below 1% in a small proportion (6.8%) of the community health workers (CHWs) involved. The lowest error rate was observed in CHWs working in Burkina Faso [64].

## 5. Conclusions

This study analyzed data on malaria cases (suspected, tested, and confirmed) from Burkina Faso’s epidemiological surveillance system (ENDOS-BF) between 2013 and 2018 and examined the potential effects of key antimalarial interventions. An increasing trend in outpatient consultations was recorded between 2013 and 2018, with an increase in confirmed malaria cases from 2016 onwards. This trend strongly coincides with the introduction of the new policy of free-of-charge health care for children younger than 5 years. The results indicate that the fraction of correctly diagnosed cases has increased and that registration (and notification) practices have improved. This calls for continued control efforts to diagnose all true cases of malaria (see the hippopotamus almost completely) in a comprehensive manner and to treat them effectively to halt the progression of cases to adverse outcomes, i.e., death. Lastly, the objective of the free healthcare policy is to decrease malaria morbidity and mortality in children under five. Regarding malaria morbidity, our results indicate, on the contrary, an increased malaria incidence. This could be explained by the relatively recent implementation of the policy and we hypothesize that a reevaluation in the long run could evidence the initially expected effect, that is, a decrease in malaria incidence. To date, the effect of the free healthcare policy on malaria mortality in children under five living in Burkina Faso has not been evaluated yet.

## Figures and Tables

**Table 1:** Effect of interventions on malaria reported cases incidence estimated from Bayesian spatio-temporal models adjusted for socio-economic and climatic factors.

Predictor ^1^	Confirmed Cases * (n = 23,634,433)	Tested Cases ** (n = 28,653,332)	Confirmed Cases ** (n = 23,634,433)
	IRR (95% BCI)	IRR (95% BCI)	IRR (95% BCI)
Free-of-charge health care			
Before 2016	1	1	
After 2016	2.078 (1.339, 3.197)	2.088 (1.378, 3.136)	2.128 (1.276, 3.518)
ITN Use	0.694 (0.463, 1.039)	0.630 (0.429, 0.925)	0.740 (0.458, 1.190)
ACT Use	0.902 (0.666, 1.22)	0.917 (0.688, 1.22)	0.865 (0.605, 1.236)
Care seeking	1.525 (1.027, 2.266)	1.524 (1.047, 2.222)	1.517 (0.951, 2.423)
Poverty	1.09 (0.843, 1.406)	1.027 (0.804, 1.312)	1.126 (0.832, 1.522)
RDT availability	1.559 (1.228, 1.978)	1.702 (1.365, 2.123)	1.523 (1.150, 2.020)
Number of health facilities			
Less than 1 per 5000 inhabitants	1	1	
More than 1 per 5000 inhabitants	1.007 (0.940, 1.080)	1.052 (0.988, 1.120)	1.005 (0.926, 1.091)
Rainfall (with lag of order 1)			
(0,85.7]	1	1	
(85.7,171]	1.013 (0.935, 1.097)	0.988 (0.918, 1.063)	1.011 (0.92, 1.111)
(171,257]	1.113 (0.984, 1.258)	1.084 (0.968, 1.213)	1.105 (0.956, 1.276)
Temperature (°C)			
(20.3,26]	1	1	
(26,31.6]	1.034 (0.988, 1.082)	1.023 (0.982, 1.067)	1.034 (0.979, 1.091)
(31.6,37.2]	1.023 (0.958, 1.092)	1.018 (0.959, 1.081)	1.021 (0.946, 1.103)
	Mean (95% BCI)	Mean (95% BCI)	Mean (95% BCI)
Spatial variance	1.004 (1.001, 1.014)	1.004 (1.00, 1.0160)	1.002 (1.000, 1.015)
Temporal variance	1.347 (1.183, 1.759)	1.303 (1.161, 1.636)	1.293 (1.162, 1.652)
Temporal correlation	2.085 (1.818, 2.38)	2.094 (1.831, 2.358)	3.735 (3.134, 5.323)
Variance of interaction effect	1.129 (1.100, 1.166)	1.123 (1.093, 1.16)	1.336 (1.289, 1.388)
Interaction effect correlation	2.635 (2.606, 2.656)	2.649 (2.625, 2.667)	2.804 (2.776, 2.849)

^1^ Coverages of ITN, ACT, care seeking, and RDT availability were modelled on a scale of 0 to 1; therefore, a one unit increase in coverage corresponds to a 100% increase, which implies a shift of the current value by 100%. * log (total population children five) has been use as offset in the Neg Bin model. ** log (total cases malaria) has been use as offset in the Neg Bin model.

## Research Data

The dataset of malaria cases aggregated at the health district level is available at the Directorate General of Studies and Sectoral Statistics of the Ministry of Health in Burkina Faso.

## Abbreviations

BCI	Bayesian credible intervals
DIC	Deviance information criterion
DHIS2	District Health Information System
INLA	Integrated Nested Laplace Approximation
ITN	Insecticide-treated Nets
IRR	Incidence rate ratio.
RDT	Rapid Diagnostic Test

## Appendix A. Estimating District-Level Interventions Coverage, Socioeconomic Status, Health-Seeking Behavior, and Availability of RDTs

Data on coverage of interventions (ITNs and ACTs), poverty, and health-seeking behaviors used in this study are from MIS 2014 and MIS 2017 and were available only at the national and regional level. Indeed, the sample of these population surveys was designed to give representative unbiased estimators at the national and regional level. A conditional autoregressive model (CAR) formulated with a binomial distribution was performed to estimate indicators at the district level.

Let $Y_{i}$ be the number of households that possessed at least one ITN in district i=1,…,70, and $N_{i},$ the total number of households sampled and interviewed in district i. We assume that $Y_{i}$ follows a Binomial distribution, that is, $Y_{i}|N_{i},\pi \left(i\right)\sim \mathrm{Bin}\left(N_{i},\pi \left(i\right)\right)$ ∀i=1,…,70, where π(i) is the proportion of households with at least one ITN in district i. A Bayesian CAR model to estimate district-level ITN coverage was formulated as follows:

$$\mathrm{logit}\left(\pi \left(j\right)\right)=\beta_{0}+\omega_{j},$$

where $\beta_{0}$ is a constant, and $\omega_{i}$, i=1,…,70, are modeled via a CAR process. Each $\omega_{i}$ conditional on the neighbor $\omega_{j}$ follows a normal distribution with a mean equal to the average of the neighboring districts, $\omega_{j}$, and variance inversely proportional to the number of neighbor districts, $n_{i}$, that is; $\omega_{i}|\omega_{j}\sim N\left(\gamma \sum_{l\in \delta_{i}}\;\omega_{j},\;\frac{\sigma_{\omega }^{2}}{n_{i}}\right)$, where γ quantifies the amount of spatial correlation present in the data, $\sigma_{\omega }^{2}$ measures the spatial variance. $\omega_{i}$ and $\omega_{j}$ are adjacent districts in the set of all adjacent districts $\delta_{i}$ of district i, and $n_{i}$ are the number of adjacent districts. Following the standard formulation of Bayesian regression models, we assumed vague priors: A non-informative Gaussian distributions with mean 0 and variance 10^2^ for $\beta_{0}$, that is, $\beta_{0}$~N(0, 10^2^). An inverse gamma prior distribution with mean 10 and variance 100 was considered for $\sigma_{\omega }^{2}$, i.e., $\sigma_{\omega }^{2}\sim Ga\left(0.1,0.001\right).$

Similar formulations were applied for ACTs, malaria treatment-seeking behavior, and household asset index; however, the latter was modeled by a first-stage Gaussian distribution. The same model was applied for the availability of RDTs in health centers using data from the SARA survey, which was also designed to be representative at the regional level.

## Appendix B. Statistical Modeling

To estimate the magnitude of spatial heterogeneity and temporal trend of malaria cases in children, a negative binomial model implemented in a Bayesian framework was designed to include environmental factors. Structured and unstructured parameters of space, time, and their interactions were taken into account.

Thus, the number of severe malaria cases, $Y_{\mathrm{ijt}},$ observed in district i=1,…,70 at time t = 1, …, 12 of year j=1,…, 6 follows a negative binomial distribution, such that:

$Y_{\mathrm{ijt}}\sim \mathrm{NB}\left(p_{\mathrm{ijt}},\;r\right)$ were $p_{\mathrm{ijt}}=r/\left(r+{µ}_{\mathrm{ijt}}\right)$, where r is the dispersion parameter and ${µ}_{\mathrm{ijt}}$ is the average monthly number of malaria cases in the district. The model is formulated as follows:

$$\log\left({µ}_{\mathrm{ijt}}\right)=\log\left(N_{ijt}\right)+\alpha +X^{T}\beta +{µ}_{\mathrm{ij}}+\upsilon_{\mathrm{ij}}+\gamma_{\left(j-1\right)*12+t}+\phi_{\left(j-1\right)*12+t}+\delta_{i\left(j-1\right)*12+t},$$

where $N_{ijt}$ is the offset district-month-specific population for malaria incidence estimation or the offset district-month-specific total cases of malaria for the estimated malaria case confirmation rate and tested rate.

α is the intercept, and β is a vector of regression coefficients associated with the vector of predictors $X_{it}$ (environment).

The $\gamma_{\left(j-1\right)*12+t}$ are monthly random effects modeled by the first-order random walk (RW(1)) with a temporal variance of $\sigma_{1}^{2}$, that is to say, $\gamma_{l}\sim \mathrm{RW}\left(1\right)$ where $\gamma_{1}\sim N\left(0,\frac{\sigma^{2}}{1-p^{2}}\right)$, $\gamma_{l}\sim N\left({\rho \gamma }_{l-1},\sigma^{2}\right)$, l = 2, …, 67, and the autocorrelation parameter, ρ, quantifies the degree of dependence between successive months.

${µ}_{\mathrm{ij}}$ + $\upsilon_{\mathrm{ij}}$ is the spatial random effect of district *i* year j.

${µ}_{\mathrm{ij}}$ are random effects specific to district i of year j, modeled by conditional autoregressive processes CAR ($\sigma_{1j}^{2})$.

Each ${µ}_{\mathrm{ij}}$ conditionally to is its neighbors ${µ}_{kj}$ follows a normal distribution with a mean equal to the average of the neighboring districts, ${µ}_{kj}$, $k\in N_{i},$ and a variance inversely proportional to the number of neighboring districts, $n_{i}$, that is to say; ${µ}_{ij}|{µ}_{kj}\sim N\left(\pi_{j}\sum_{k\in \delta_{i}}\;{µ}_{kj},\;\frac{\sigma_{2j}^{2}}{n_{i}}\right),$ where $\pi_{j}$ quantifies the degree of spatial correlation present in the data of the year j, and $\sigma_{2j}^{2}$ measures the spatial variance. ${µ}_{ij}\;\mathrm{and}$ ${µ}_{kj}$ are adjacent districts in all of the adjacent districts $N_{i}$ of district i, and $n_{i}$ is the number of adjacent districts.

$\upsilon_{\mathrm{ij}}$ are random effects exchangeable between the year and district, that is to say $\upsilon_{\mathrm{ij}}\sim N\left(0,\sigma_{2j}^{2}\right)$.

$\phi_{\left(j-1\right)*12+t}$ represent the unstructured temporal effect and follows a normal distribution $N\left(0,\frac{1}{\tau_{\phi }}\right).$

$\delta_{i\left[\left(j-1\right)*12+t\right]}$ represents the spatio-temporal interaction and essentially reflects any deviation from the main spatial and temporal effects. They follow a Gaussian distribution with a precision matrix given by $\tau_{\delta }R_{\delta }$, where $\tau_{\delta }$ is an unknown scalar while $R_{\delta }$ is the structure matrix, identifying the type of temporal and/or spatial dependence between the elements.

β is a non-informative normal prior distribution, r~Gamma(1,100)

A is a non-informative inverse gamma priori distribution for $\sigma_{1j}^{2},\;\sigma_{2j}^{2}\;and\;\sigma^{2}$, i.e., $\sigma_{1j}^{2},\;\sigma_{2j}^{2},\sigma^{2}\sim Ga\left(0.1,0.001\right),\;j=1,\ldots ,\;6$ and a uniform posterior distribution for ρ, i.e., ρ~U[−1,1].
